# Liver Biochemical Abnormalities in Adolescent Patients with Turner Syndrome

**DOI:** 10.4274/jcrpe.galenos.2019.2018.0271

**Published:** 2019-11-22

**Authors:** Małgorzata Wójcik, Anna Ruszała, Dominika Januś, Jerzy B. Starzyk

**Affiliations:** 1 Jagiellonian University Medical College, Pediatric Institute, Department of Pediatric and Adolescent Endocrinology, Chair of Pediatrics; Children’s University Hospital, Kraków, Poland; ★ Contributed equally to this work

**Keywords:** Turner syndrome, children, liver, estrogen

## Abstract

**Objective:**

Elevated liver function tests (LFTs) are common in adult Turner syndrome (TS) patients. Data regarding children and adolescents are lacking. To investigate the prevalence of abnormal LFTs in children and adolescents with TS during several years of observation; to evaluate the potential impact of increased body mass index (BMI) and sex hormone replacement therapy (HRT) on LFTs.

**Methods:**

The analysis included 100 girls with TS, aged 4-16 years, all of whom were receiving recombinant human growth hormone therapy. A longitudinal study was conducted which included 81 patients.

**Results:**

Mean BMI-standard deviation (SD) score of the subjects was 0.63 (SD: 1.53). Forty-four were being treated with HRT. Elevated LFTs were found in 34% of the patients overall (32% not receiving HRT vs 36% on HRT). The relative risk of increased LFTs was not higher in obese vs normal weight [odds ratio (OR): 0.2; 95% confidence interval (CI): 0.1-0.36, p=0.38 vs OR: 0.16; 95% CI: 0.08-0.3, p=0.1]. HRT did not increase the risk of abnormal LFTs activity (OR: 0.8; 95% CI: 0.5-1.2, p=0.37 vs OR: 0.7; 95% CI: 0.4-1.1, p=0.27). During the follow-up period (mean±SD=4.31±0.82 years), no patient developed overt liver disease. There was no significant increase nor decrease of abnormal LFT frequency in the subsequent years of follow up.

**Conclusion:**

Constantly elevated LFTs in TS are common in children and adolescents with TS. However the causes and clinical significance remain unclear. This study suggests that obesity and HRT do not increase the risk of elevated LFTs.

**What is already known on this topic?**

Elevated liver function tests (LFTs) are common in adult patients with Turner syndrome (TS). Potential causes and mechanisms suggested in the literature are not clear, and may include autoimmunity, venous malformations, obesity and sex hormones replacement therapy (HRT).

**What this study adds?**

Elevated LFTs are common in children and adolescents with TS. Obesity and HRT do not increase the risk of elevated LFTs.

## Introduction

Turner syndrome (TS) affects approximately 1 per 2500 live female births and is one of the most common chromosomal aberration in females (1,2). It is caused by a partial or complete X chromosome monosomy. Conditions often seen in TS include: short stature, ovarian dysgenesis, dysmorphic features and endocrine disturbances such as diabetes mellitus and thyroiditis. Liver involvement indicated by abnormal liver function tests (LFT) seems to be frequent in adult TS patients, with a prevalence of 20 to 80% (3,4,5,6). Data on children and adolescents are lacking. The causes and clinical significance of this phenomenon are unclear. Nevertheless, overt liver diseases are also more common in TS patients than in the general population. The hepatic histological changes reported in TS patients vary and include minimal abnormalities, steatosis, steatohepatitis, biliary involvement, nodular regenerative hyperplasia and even cirrhosis (5,6,7,8,9,10,11,12,13,14,15,16,17,18,19). Potential causes and mechanisms are not clear and may include autoimmune processes, venous malformations, obesity and sex hormone replacement therapy (HRT) (5,6,7,8,9,10,11,12,13,14,15,16,17,18,19,20). However, some basic and animal studies point to the crucial a role of estrogen deficiency or estrogen receptor malfunction in the development of liver impairment (21,22,23,24,25,26,27).

The aims of this study were to investigate the prevalence of abnormal LFTs in children and adolescents with TS; to analyse LFTs changes and their clinical significance over several years of observation; to evaluate the potential impact of increased body mass index (BMI) and sex HRT on LFTs.

## Methods

The analysis included 100 girls with TS, aged 4-16, all of whom were being treated with human recombinant growth hormone. 44 patients were on HRT-estrogen and estrogen/progestin patches. Patients were treated with daily injections of human rekombinant growth hormone, dose 0.33-0.47 mg/kg/week. It was a retrospective analysis plus prospective follow-up period. Blood was collected in the fasting state, in the morning (7.00-9.00), during routine examinations performed in patients with TS.

The activity of aspartate aminotransferase (AST) and alanine aminotransferase (ALT) was measured in fresh serum samples using dry chemistry (VITROS^®^ 5.1 FS, Ortho Clinical Diagnostics).

Body height and weight were measured to the nearest 0.1 cm and 0.1 kg, respectively, using a stadiometer (Harpenden, UK) and a balanced scale (SECA).

### Statistical Analysis

To compare the two sets of data, Student’s t-test or two-sided Mann-Whitney U test were used. For a correlation analysis, the correlation coefficient (R) and regression analysis were used. Odds ratio (OR) was calculated using logistic regression analysis. A probability value of less than 0.05 was accepted to be statistically significant.

### Ethics

The investigation was conducted according to the principles expressed in the Declaration of Helsinki. The participants and/or their parents signed informed consent. The study has been approved by the Jagiellonian University Bioethical Committee (decision number: KBET/102/B/2012);

## Results

The longitudinal study included 81 patients (mean follow-up period: 4.31 years, SD: 0.82). Mean BMI-standard deviation (SD) score (SDS) was 0.63 (SD: 1.53).

In the whole group of patients 17 were diagnosed with obesity (9 without HRT and 8 with HRT).

Elevated LFTs were found in 34 patients 34% [in 18 (32%) without HRT *vs* in 16 (36%) on HRT]. Increased AST activity was present in 10 (18%) without HRT; in 5 (11%) on HRT), and elevation of ALT [in 9 (16%) without HRT and in 11 (25%) on HRT]. The mean values of AST in both groups (without HRT and with HRT) were 42.7 IU/L and 44.2 IU/L p=0.8, and the mean value of ALT were 27.5 IU/L and 29.9 IU/L, p=0.14 respectively.

The mean values of AST in patients with obesity and non obese were 47.5 IU/L and 42.5 IU/L, p=0.8. The mean values of ALT in patients with obesity and non obese were 35.6 IU/L and 27 IU/L, p=0.037.

**Figure 1:**
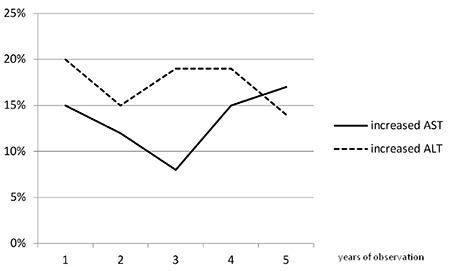
Percentage of abnormal results of liver function tests during subsequent years of observation AST: aspartate aminotransferase, ALT: alanine aminotransferase

The relative risk of increased LFTs activity was not higher in obese *vs* normal weight [OR: 0.2; 95% confidence interval (CI): 0.1-0.36, p=0.38 *vs* OR: 0.16; 95% CI: 0.08-0.3, p=0.1]. HRT did not increase the risk of abnormal LFTs activity (OR: 0.8; 95% CI: 0.5-1.2, p=0.37 *vs* OR: 0.7; 95% CI: 0.4-1.1, p=0.27). During the follow-up period, no patient developed overt liver disease. There was no significant increase nor decrease of the abnormal LFTs frequency in the subsequent years of follow up (p>0.05) (Figure 1).

## Discussion

The reported frequency of elevated LFT activity in TS patients ranges from 20 to 80%, with the highest proportion in older patients (3,4,5,6,27,28). In a recent large study (842 pediatric patients) only 3.4% of 698 examined were found to have abnormal LFT results and in patients younger than 10 years this was only found in five patients (29). Research carried out in older age groups indicates a much more frequent occurrence of abnormal LFTs. In the study of El-Mansoury et al (27) 36% of 218 adult TS patients presented with abnormal levels of one or more liver enzymes at the beginning, and subsequently 23% more developed abnormal LFTs during a 5-year follow-up. In our study, we found a similar proportion of young TS patients with elevated LFTs at the beginning of the study (34%), but we did not observe any progression during the follow-up period. Although liver disease in patients with TS is generally more common than in the general population, so far no direct correlation has been found between the development of liver disease and the occurrence of abnormal LFTs in the preceding period. In most published studies LFTs did not progress to overt liver disease (1,17,18,19). Also, little is known about the factors predisposing to abnormal LFTs. The literature suggests the possible participation of obesity and HRT by analogy to the results of research conducted in various groups of patients (4). As women with TS have short stature and abnormal body proportions, they are more likely to be overweight and obese (4,17,18,19,30). In our present study, no relationship was found between obesity and LFTs. The relative risk of the development of LFTs was comparable in patients with obesity and normal BMI-SDS. This finding is in accordance with some earlier studies in this field, which confirmed obesity as a frequent finding in TS patients, but without correlation to liver impairment (5,27,31). Another potential factor widely considered in older publications as a cause of hepatotoxicity is estrogen replacement therapy (32,33). Estrogen receptors are expressed in the liver and estrogens probably play an important role in hepatic lipid homeostasis (34,35). Despite many studies performed in this field, the causative role of estrogens is not well established. Some reports suggest that estrogen replacement therapy in TS patients can cause deterioration of liver function and in some patients discontinuation of therapy was followed by a decrease in enzyme levels (36). In contrast, some more recent studies point to a potential role of estrogen replacement as a favourable factor improving liver function (21). Although some studies reported alterations in LFTs in TS patients treated with estrogens, these alterations did not improve with the discontinuation of replacement therapy (13,20). More recent studies also found elevated LFTs in young patients before HRT, and some showed a beneficial impact of estrogen replacement (4,28,31). In our study, sex HRT did not increase the risk of elevated ALT and AST. As we examined a group of pediatric TS patients, it can be difficult to compare our results with studies based on results of adult TS patients. More recent observational studies, conducted in post-menopausal women without HRT revealed an increased risk of liver steatosis, in comparison to pre-menopausal women (21,37,38). For this reason, the importance of estrogen in liver function has become the subject of many experimental studies. A number of basic and animal studies have revealed a crucial role of estrogens and estrogen receptor deficiency in the pathogenesis of liver dysfunction. Estrogens can mediate their biologic effects in the liver through a number of mechanisms. The classic mechanism involves its binding to the steroid nuclear hormone receptors, α or β. Both have the classic features of steroid hormone receptors (39). Estrogens can also alter cell signaling via estrogen receptor α or β, localized in the cell membrane. In addition to membrane localized α and β receptors, estrogens can signal through another cell surface receptor, the G-protein coupled estrogen receptor (GPER, also called Gpr30) which is expressed in multiple tissues, including liver (40). It has been shown recently that the loss of receptor α in the liver is associated with hepatic steatosis and inflammation, and its gene expression is lower in patients with non-alcoholic steatohepatitis (41). Zhu et al (22,23) reported that estrogen treatment may reverse aspects of pathway-selective insulin resistance by promoting insulin action on glucose metabolism but limiting hepatic lipid and diacylglycerol deposition. Estrogen treatment reduces liver fat storage on several levels, mainly by blocking insulin signaling to liver acetyl-CoA carboxylase and reducing hepatic apoB100 and phospholipid transfer protein. This protective effect of estrogen treatment requires intact hepatic estrogen signaling through estrogen receptor α. By contrast, hepatic estrogen signaling may not be required for the effects of estrogen treatment on body weight and adiposity (22,23). Moreover, Kao et al (42) found that estrogen receptor α could be an important mediator of liver regeneration. What is more, it has been shown that estrogen receptor β agonist might provide therapeutic benefits in liver steato-hepatititis by directly modulating the bile acid receptors in the liver, which have important functions in the liver, and indirectly, by inhibiting adiposity (43). The mechanisms by which estrogen signaling protects against hepatic steatosis also include reductions in de novo lipogenesis, as reported by Gao et al (25). These mechanisms may be helpful for understanding mechanisms of liver impairment in TS patients and the favourable action of estrogen replacement.

### Study Limitations

The main limitation is its retrospective character leading to a lack of long-term observation for the whole group. Due to different models (transdermal/oral) of HRT and various estradiol doses, the effect of estrogens on LFTs could not be accurately analyzed.

## Conclusion

Constantly elevated LFTs in TS are common in children and adolescents with TS. However the causes and clinical significance remain unclear. This study suggests that obesity and HRT do not increase the risk of elevated LFTs.
